# Supplementary material for: Quantitative Risk Assessment of Five Foodborne Viruses in Shellfish Based on Multiplex qPCR
Source: Foods. 2023 Sep 17;12(18):3462. doi: 10.3390/foods12183462 (PMC10530164; doi:10.3390/foods12183462)
Supplement: Supplementary file 1 [file foods-12-03462-s001.zip › foods-2609015-supplementary.pdf]

Quantitative risk assessment of five foodborne viruses in shellfish based on multiplex RT-qPCR

Zhendi Yu [1], Zhangkai Xu [1,2], Jiang Chen [1,3], Lili Chen [4], Ningbo Liao [4,5], Ronghua Zhang [4,*] and Dongqing Cheng [1,*]

[1] School of Medical Technology and Information Engineering, Zhejiang Chinese Medical University, Hang-zhou 310053, China
[2] Department of Clinical Laboratory, Zhejiang Hospital, Hangzhou 310013, China
[3] Department of Laboratory Medicine, The First Affiliated Hospital, Zhejiang University School of Medicine, Hangzhou 310003, China
[4] Department of Nutrition and Food Safety, Zhejiang Provincial Center for Disease Control and Prevention, Hangzhou 310051, China
[5] College of Food Science and Engineering, Jiangxi Agricultural University, Nanchang 330045, China
[*] Correspondence: chengdq@zcmu.edu.com (D. C.); rhzhang@cdc.zj.cn (R. Z.)

Table S1. Table of six shellfish collection samples. Samples of six common shellfish species, including commercially available *Razor Clam, Scallop, Clam, Oyster, Venerupis, and Mussel,* were collected from January 2019-December 2019, and sampling was conducted once a month. A total of 103 fresh shellfish samples were collected, of which 29 were collected in spring (March-May), 30 in summer (June-August), 18 in autumn (September-November) and 26 in winter (December-February).

	Spring	Summer	Autumn	Winter	Total
Razor Clam	5	5	3	4	17
Scallop	5	5	3	6	19
Clam	5	5	3	4	17
Oyster	5	5	3	4	17
Venerupis	4	5	3	3	15
Mussel	5	5	3	5	18
Total	29	30	18	26	103

Table S2: Shellfish intake and frequency in the population (Zhao et al.,2021).

	Shellfish intake (g per meal)	Frequency of shellfish intake (times per year)
Gender		
Male	38.4	21.1
Female	34.3	15.6
Mean value	36.2	18.3
Age		
0-4	6.6	8.7
5-18	33.2	12.8
19-64	48.9	23.8
≥65	23.7	13.8

Table S3: The virus infection risk classification matrix (Hernandez-Jover et al.,2021).

Probability level (score)	Hazard level (score)				
	Minimal risk (1)	Low risk (2)	Medium risk (3)	high risk (4)	Extremely high risk (5)
<1% (1)	1	2	3	4	5
1% ~ <5% (2)	2	4	6	8	10
5% ~ <10% (3)	3	6	9	12	15
10% ~ <20% (4)	4	8	12	16	20
>20% (5)	5	10	15	20	15

Probability level is the probability of disease per meal for QMRA or per day per person for risk ranger.

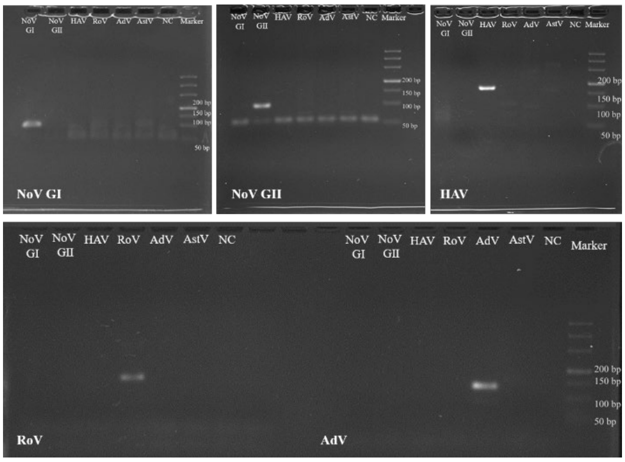

Figure S1: Specificity of detection for the multiplex qPCR method. The primer-probe pairs of each foodborne virus were verified by crossover experiments (Lee et al.,2018). The established PCR method was used to detect Norovirus GⅠ, Norovirus GⅡ, Hepatitis A virus, Adenovirus, Rotavirus standard products, and Astrovirus respectively. The specific strips were identified by 2% agarose gel electrophoresis. The specificity of the method was evaluated. The multiplex RT-qPCR assay was used to amplify other common foodborne viruses, including Norovirus types GI, GII, Hepatitis A virus, Rotavirus, Adenoviruses and Astrovirus. As speculated, only strong fluorescence signals were obtained for norovirus

types GI, GII, Hepatitis A virus and Rotavirus. The signals for other samples and negative controls were below the baseline detection levels, indicating that the multiplex real-time PCR method has good specificity.

Table S4: Detection limit results of the One-Step Single and Multiplex PCR. To compare the detection limits of the one-step single and multiplex RT-qPCR methods, 10-fold serial dilutions (10^5 to 10^1 copies/μL) of five different viral RNA samples in DEPC-treated water were tested. In the one-step single RT-qPCR, the PCR mixture conditions were the same as multiplex RT-qPCR except that the specific primer set for each target virus was contained in a separate reaction tube. Single and multiplex RT-qPCR methods were performed using the same dilution series, the same PCR machine, and the same thermal cycling conditions. The detection limits of the one-step single and multiplex RT-PCR were both seen at the 10^1 copies/μL for NoV GI, NoV GII and HAV, and at the 10^2 copies/μL for RoV.

Ct value	10^5 copies/μL		10^4 copies/μL		10^3 copies/μL		10^2 copies/μL		10^1 copies/μL	
	Single RT-PCR	Multiplex RT-PCR	Single RT-PCR	Multiplex RT-PCR	Single RT-PCR	Multiplex RT-PCR	Single RT-PCR	Multiplex RT-PCR	Single RT-PCR	Multiplex RT-PCR
NoV GI	21.47	21.75	25.72	25.63	29.13	29.36	32.68	32.98	34.53	35.67
NoV GII	21.36	21.92	25.58	25.89	29.13	29.16	33.31	33.47	35.75	36.15
HAV	17.17	18.12	21.79	22.23	25.72	25.85	29.64	29.23	31.79	32.67
RoV	25.53	25.96	28.61	29.45	32.68	32.97	34.75	35.89	ND	ND
AdV	28.14	28.26	31.47	31.95	35.39	35.78	38.07	38.21	ND	ND

Table S5: Repeatability test of the multiplex qPCR. The intra-assay CVs were evaluated at the concentration of 1×10^6 to 1×10^9 copies/μL, respectively. The results showed that the intra-assay CV were below 1.72%. Therefore, the multiplex real-time PCR assay developed in this study is reliable and accurate.

	viruses	Concentration (copies/μL)			
		1×10^9	1×10^8	1×10^7	1×10^6
($\bar{x}$±s)	NoV-GI	13.65±0.11	16.39±0.11	22.72±0.14	26.38±0.09
	NoV-GII	15.40±0.11	20.84±0.11	24.91±0.10	28.26±0.39
	HAV	16.42±0.28	19.08±0.29	22.11±0.25	27.90±0.04
	RoV	11.29±0.16	15.36±0.09	19.89±0.18	22.73±0.09
	AdV	19.79±0.06	21.85±0.12	24.38±0.12	25.31±0.18
CV (%)	NoV-GI	0.83	0.67	0.61	0.35
	NoV-GII	0.71	0.53	0.39	1.38
	HAV	1.72	1.53	1.13	0.16
	RoV	1.41	0.58	0.93	0.41
	AdV	0.32	0.55	0.48	0.73

Table S6: Number of shellfish samples with multiple viruses detected. Among the 39 positive samples, 7 samples had multiple foodborne viruses detected simultaneously, of which 1 sample had both NoV GI, NoV GII and RoV, 2 samples had both RoV and HAV, 3 samples had both NoV GI and NoV GII, 1 sample had both NoV GI and RoV, and 1 sample had both NoV GI and RoV.

Virus detection	Number
NoV GⅠ + NoV GⅡ + RoV	1
HAV + RoV	2
NoV GⅠ + NoV GⅡ	3
NoV GⅠ + RoV	1

References

Hernandez-Jover M., Culley F., Heller J., Ward M.P., Jenson I., 2021. Semi-quantitative food safety risk profile of the Australian red meat industry. Int J Food Microbiol. 353, 109294.

Lee S.Y., Kim M.J., Kim H.J., Jeong K.C., Kim H.Y., 2018. Simultaneous Detection of Four Foodborne Viruses in Food Samples Using a One-Step Multiplex Reverse Transcription PCR. J Microbiol Biotechnol. 28, 210-217.

Zhao F., Ding G., Wang S., Cai Y., Xu J., 2021. Preliminary Quantitative Risk Assessment of Norovirus in Shellfish in the Yellow Sea and Bohai Sea of China. Foodborne Pathog Dis. 18, 668-674.
